# Crystal Structure of *Sus scrofa* Quinolinate Phosphoribosyltransferase in Complex with Nicotinate Mononucleotide

**DOI:** 10.1371/journal.pone.0062027

**Published:** 2013-04-23

**Authors:** Hyung-Seop Youn, Mun-Kyoung Kim, Gil Bu Kang, Tae Gyun Kim, Jung-Gyu Lee, Jun Yop An, Kyoung Ryoung Park, Youngjin Lee, Jung Youn Kang, Hye-Eun Song, Inju Park, Chunghee Cho, Shin-Ichi Fukuoka, Soo Hyun Eom

**Affiliations:** 1 School of Life Sciences, Gwangju Institute of Science & Technology, Gwangju, Republic of Korea; 2 Stetiz Center for Structural Biology, Gwangju Institute of Science & Technology, Gwangju, Republic of Korea; 3 School of Culture and Creative Studies, Aoyama Gakuin University, Tokyo, Japan; Monash University, Australia

## Abstract

We have determined the crystal structure of porcine quinolinate phosphoribosyltransferase (QAPRTase) in complex with nicotinate mononucleotide (NAMN), which is the first crystal structure of a mammalian QAPRTase with its reaction product. The structure was determined from protein obtained from the porcine kidney. Because the full protein sequence of porcine QAPRTase was not available in either protein or nucleotide databases, cDNA was synthesized using reverse transcriptase-polymerase chain reaction to determine the porcine QAPRTase amino acid sequence. The crystal structure revealed that porcine QAPRTases have a hexameric structure that is similar to other eukaryotic QAPRTases, such as the human and yeast enzymes. However, the interaction between NAMN and porcine QAPRTase was different from the interaction found in prokaryotic enzymes, such as those of *Helicobacter pylori* and *Mycobacterium tuberculosis*. The crystal structure of porcine QAPRTase in complex with NAMN provides a structural framework for understanding the unique properties of the mammalian QAPRTase active site and designing new antibiotics that are selective for the QAPRTases of pathogenic bacteria, such as *H. pylori* and *M. tuberculosis*.

## Introduction

Nicotinamide adenine dinucleotide (NAD^+^) is an essential cofactor related to energy metabolism and is also involved in signal transduction [1], [2]. The electron-transferring properties of NAD^+^ and its function as cofactor in multiple redox reactions are well known. NAD^+^ is also a precursor of intracellular calcium-mobilizing agents, such as cyclic ADP-ribose (cADPR) and nicotinate adenine dinucleotide phosphate. Even the redox state of NAD^+^ might directly influence transcriptional pathways involved in development, cell cycle regulation, and transformation [3].

NAD^+^ biosynthesis is essential for all organisms. NAD^+^ is synthesized via two pathways in eukaryotic and some prokaryotic organisms. One is a *de novo* pathway that is related to tryptophan degradation [4]. The other, the salvage pathway, occurs through the recycling of degraded NAD^+^ products, such as nicotinamide. In *de novo* NAD^+^ biosynthesis, the two precursors quinolinate (QUIN) and nicotinate receive a phosphoribosyl moiety from 5-phosphoribosyl-1-pyrophosphate (PRPP) via the respective phosphoribosyltransferases. The resulting nicotinate mononucleotide (NAMN) is then converted into the dinucleotide nicotinate adenine dinucleotide (NAAD). Finally, NAAD is amidated to NAD^+^. Quinolinate phosphoribosyltransferase (QAPRTase) is an essential enzyme in the first step of NAD^+^ biosynthesis, catalyzing the transfer of the phosphoribosyl moiety from PRPP to QUIN to generate NAMN.

QAPRTases have drawn attention for a specific set of properties. (1) This enzyme is used for the synthesis of defensive pyridine alkaloids in *Nicotiana* [5]. (2) A dysfunction of quinolinate metabolism in the human brain has been postulated to be involved in the pathogenesis of neurodegenerative disorders (e.g., epilepsy, Alzheimer’s disease, and Huntington’s disease). High QUIN levels can be catabolized in the brain through decarboxylation and conjugation with PRPP to form NAMN, which is catalyzed by QAPRTase [6]. (3) Some pathogens disrupt the salvage pathways, allowing the recycling of NAD^+^ through degradation to nicotinate followed by the conversion of nicotinate to NAMN by nicotinate phosphoribosyltransferase (PncB) [4]. Thus, the *de novo* pathway of NAD^+^ biosynthesis may be a possible target for antibacterial drug design [7], [8].

QAPRTase has been isolated from several sources, including *Salmonella typhimurium*, a soil pseudomonad, caster beans, porcine liver, rat liver and brain, and human liver and brain [9]–[14]. QAPRTase is reported to exist as a dimer or a hexamer depending on the source. Structural studies of QAPRTase from *Mycobacterium tuberculosis*, *Salmonella typhimurium*, and *Thermotoga maritima* have shown that the active enzyme exists as a dimer, which is essential for full activity [7], [8], [15]. In *Homo sapiens*, *Rattus norvegicus*, and *Sus scrofa*, QAPRTases were reported to exist as hexamers [10], [12], [13]. Although several QAPRTase structures have been determined as a result of extensive crystallographic studies, structural information regarding the reaction mechanism of QAPRTase in higher eukaryotes, including mammals, is limited due to the lack of a structure in complex with a reactant or product.

Here, we report the 2.1 Å resolution crystal structure of *Sus scrofa* QAPRTase (*Ss*-QAPRTase) in complex with NAMN. Our results represent the first crystal structure of a mammalian hexameric QAPRTase with its reaction product and may provide structural information useful for understanding the mode of binding of NAMN with eukaryotic QAPRTases and for designing drugs specifically targeting the QAPRTases of pathogenic bacteria rather than those of mammals.

## Materials and Methods

### Ethics Statement

Committee approval of Animal Care and Use was not obtained for this study because porcine tissues were taken from the authorized slaughterhouse (SAMHO Co. Ltd., registration number 409-81-43369, Republic of Korea). We have obtained permission for experimental purpose from this slaughterhouse to use porcine tissues. Animals were carefully protected and tissue extraction was performed with qualified veterinarian.

### Amino Acid Sequence Determination

The amino acid sequence of full-length *Ss*-QAPRTase is not currently available in any sequence database. To determine the amino acid sequence of *Ss*-QAPRTase, complementary DNA (cDNA) of *Ss*-QAPRTase was synthesized by the reverse transcriptase-polymerase chain reaction (RT-PCR). Total RNA was isolated from 4 different lobes (left, right, caudate, and quadrate) of the porcine liver using TRI reagent (Molecular Research Center) and reverse transcribed into cDNA using Omniscript Reverse Transcriptase (Qiagen). The cDNA was amplified by PCR with primers selected from the regions of the *Ss*-QAPRTase sequence that are conserved among mammalian species (Table S1), and the PCR products were analyzed by automated DNA sequencing (Macrogen). The nucleotide sequence data of *Ss*-QAPRTase has been submitted to the NCBI GenBank with the accession number KC185402.

### Protein Purification and Crystallization

The purification and crystallization of *Ss*-QAPRTase from the porcine kidney have been described elsewhere [16], [17]. Briefly, frozen porcine kidney was homogenized in 50 mM potassium phosphate, pH 7.0, containing 10 mM β-mercaptoethanol (standard buffer). The supernatant of the crude extract went through (NH_4_)_2_SO_4_ fractionation followed by anion exchange using a DEAE-Sephadex A-50 column. The sample was eluted with 50–500 mM potassium phosphate, pH 7.0, containing 10 mM β-mercaptoethanol. Fractions were pooled, and the protein was precipitated using 60% (NH_4_)_2_SO_4_ and dissolved in standard buffer. The solution was dialyzed with 50 mM Tris–HCl, pH 8.5, containing 130 mM sodium citrate and loaded onto a Superdex-200 16/60 column (Pharmacia) equilibrated in 20 mM HEPES–NaOH, pH 7.5, 100 mM KCl. Fractions containing oligomeric *Ss*-QAPRTase were pooled and concentrated to 15 mg ml^–1^ (Figure S1). The *Ss*-QAPRTase was cocrystallized with NAMN at room temperature (294±1 K) using the hanging-drop vapor-diffusion method. NAMN was supplemented into the reservoir solution (5 mM, 11 times excess than the protein). *Ss*-QAPRTase (15 mg ml^–1^) in 20 mM HEPES–NaOH, pH 7.5, and 100 mM KCl was mixed with an equal volume of reservoir solution consisting of 100 mM Tris–HCl, pH 8.0, 16–24% (w/v) PEG 8000, 150–200 mM ammonium acetate, and 5 mM NAMN. Rod-shaped single crystals were grown to maximal dimensions of 0.3×0.1×0.1 mm over the course of a week.

### Data Collection and Structure Determination

For data collection, the *Ss*-QAPRTase–NAMN cocrystal was transferred to cryoprotectant containing 100 mM Tris–HCl, pH 8.0, 16–24% PEG 8000, 150–200 mM ammonium acetate, and 20% (v/v) ethylene glycol and flash frozen in a liquid nitrogen stream at 95 K. The X-ray diffraction data were collected on the 18B beamline at the Photon Factory (Tsukuba, Japan). The data set was processed and scaled with HKL2000 [18]. The *Ss*-QAPRTase–NAMN cocrystal diffracted to 2.1 Å and belongs to the *P*321 space group with cell dimensions *a* = *b* = 119.1, *c* = 93.7 Å, *γ* = 120.0°. The Matthews coefficient was calculated to be 3.10 Å^3^ Da^–1^, which correspond to a solvent content of 60.3% assuming two molecules in the asymmetric unit [19]. The L test for twinning [20] indicated that the data were perfectly twinned with L-statistics of 0.38. The structure was solved via molecular replacement with PHASER [21] using the dimeric structure of human QAPRTase (PDB ID: 2JBM) as the search model. Although the asymmetric unit contains two subunits of *Ss*-QAPRTase, generation of crystallographic symmetry-related molecules showed that the biological unit of the enzyme is a hexamer. Moreover, the hexameric structure of *Ss*-QAPRTase is consistent with that of other eukaryotes, including the human and yeast structures (PDB ID: 2JBM and 3C2E). Two NAMN molecules obtained from the RCSB Ligand Expo (http://ligand-expo.rcsb.org/pyapps/ldHandler.py?formid=cc-index-search&target=ncn&operation=ccid) were added into the initial model. The model including the water molecules was built using COOT [22] and refined with TLS, restrained, and amplitude-based twin refinement using REFMAC5 [23]. Further refinement using XYZ coordinates, Real-space, Rigid body, and Individual B-factor steps with phenix.refine [24] provided the final model having *R*_work_ and *R*_free_ of 21.5 and 25.9%, respectively. The atomic coordinates and structure factor for the *Ss*-QAPRTase–NAMN have been submitted to the Protein Data Bank (PDB) with the accession number 4I9A. All molecular graphics were prepared with PyMol version 1.5.0.4 [25].

## Results and Discussion

### Complementary DNA and Amino Acid Sequence Analysis

The comparison between cDNA of the *Ss*-QAPRTase used in this study and the reference sequence from *Sus scrofa* genomic DNA deposited recently (GenBank ID: NW_003534422.2) revealed that seven nucleotides are different (sequence identity = 857/864, 99%), which causes one amino acid difference (R91Q). Despite of the possibility of sequencing error or single nucleotide polymorphism in our cDNA or genomic DNA, Arg91 in our cDNA sequence is more possible because QAPRTase sequences from other mammals are conserved to arginine rather than glutamine (Figure S2). Meanwhile, seven amino acids are different between *Sus scrofa* sequence used in this study and human QAPRTase sequence, identity and homology of which are 89 and 93%, respectively (Figure S3). In spite of this difference, the residues in the NAMN binding site are totally conserved among *Ss-*QAPRTases encoded by two different DNA sequences and the human enzyme.

### Overall Quality of the Model

The three-dimensional structure of the *Ss*-QAPRTase (residues 1–288, 33 kDa) in complex with NAMN was solved by molecular replacement at 2.1 Å resolution. The data collection and refinement statistics are summarized in Table 1. The final model was refined from perfectly hemihedrally twinned crystals with a twin fraction of 0.44. To overcome the twinning problem, amplitude-based twin refinement was used in REFMAC [23]. The crystallographic R-factor of the final model is 21.5%, and the free R-factor is 25.9%. The majority of the residues (97.9%) of the *Ss*-QAPRTase model were in the favored region of the Ramachandran plot. The crystal of *Ss*-QAPRTase belongs to the space group *P*321, and a dimer is present in the asymmetric unit. However, the enzyme was purified in the hexameric form and displayed the same hexameric configuration as the crystal structure of the human QAPRTase when symmetry-related molecules were observed [26]. The stereochemistry of the models was assessed with the program PROCHECK [27]. The model of *Ss*-QAPRTase was built based on the amino acid sequence obtained by RT-PCR.

**Table 1:** Data collection and refinement statistics.

**Data collection statistics**		
X-ray source	PF-18B	
Wavelength (Å)	1.0000	
Space group	*P*321	
Unit cell parameters (Å, °)	*a* = *b* = 119.1, *c* = 93.7, *γ* = 120	
Resolution range (Å)	50–2.1 (2.14–2.10)	
Observed reflections	264,166	
Unique reflections	80,365	
Multiplicity	3.1 (3.0)	
Completeness (%)	99.5 (100.0)	
*R*_merge_ a(%)	6.4 (36.1)	
*I/*σ(*I*)	15.5 (3.3)	
**Structure phasing (Phaser)**		
TFZ	28.9	
LLG	2602	
**Refinement statistics**		
Resolution range (Å)		35–2.1
*R*_work_ b (%)	21.5	
*R*_free_ (%)	25.9	
No. of residues	576	
Hetero groups	2 NAMN	
Water molecules	128	
Average B factor (Å^2^)		
Subunits A, B	32.3, 32.9	
Solvent	38.2	
**RMSD from ideal geometry**		
RMSD bond length (Å)	0.009	
RMSD bond angle (°)	1.4	
Ramachandran statistics		
Most favored (%)	97.9	
Allowed (%)	2.1	

PF-18B, Photon Factory 18B beamline; RMSD, root-mean-square deviation.

a *R*_merge_ = ∑*_h_* ∑*_i_* | *I*(*h*)*_i_* − <*I*(*h*)> | /∑*_h_*∑*_i_I*(*h*)*_i_*, where *I*(*h*) is the intensity of reflection *h*, Σ*_h_* is the sum over all reflections, and Σ*_i_* is the sum over *i* measurements of reflection *h*.

b *R*_work_ = ∑*_hkl_*||F*_o_*|−|F*_c_*||/∑*_hkl_*|F*_o_*|; 5% of the reflections were excluded for the *R*_free_ calculation.

### Overall Structure and Hexamer Organization

**Figure 1:**
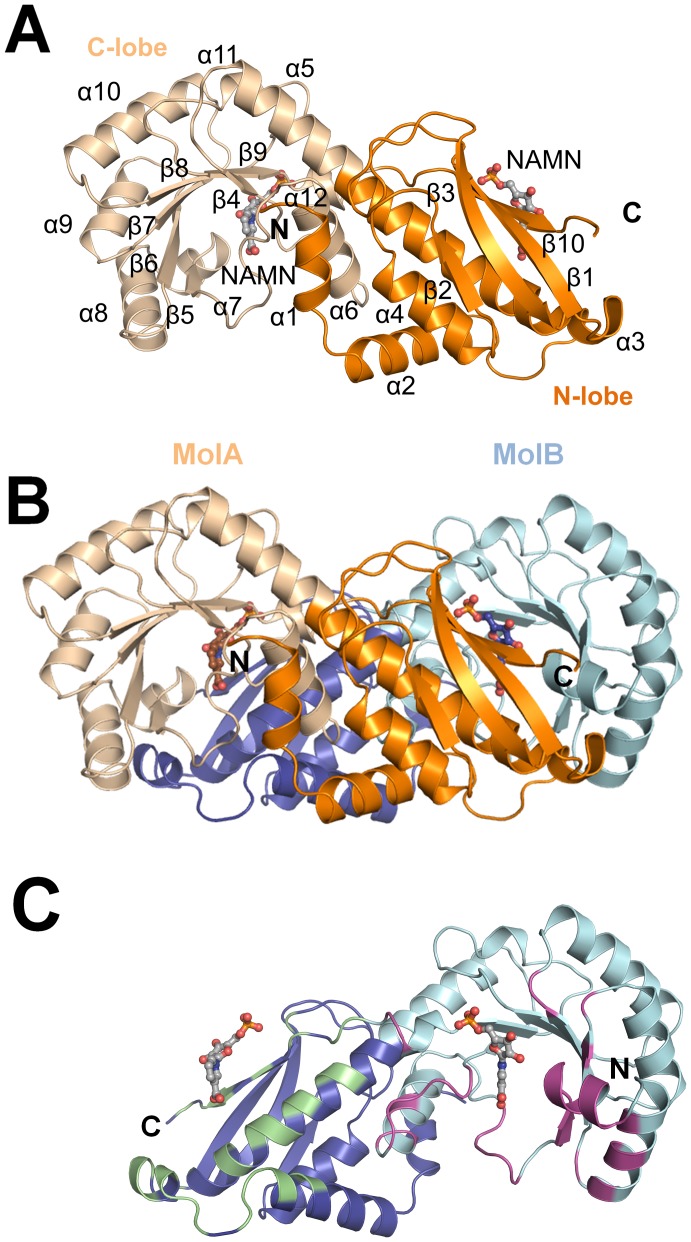
Overall structure of *Ss*-QAPRTase–NAMN complex. (A) Monomer structure. NAMN is shown as gray sticks. The N- and C-lobes are shown in orange and wheat, respectively. (B) *Ss*-QAPRTase dimer structure. The other subunit is displayed in blue. (C) Dimeric interface. The residues interacting with N- and C-lobes of the other subunit are colored in purple and green, respectively.

The monomer of *Ss*-QAPRTase comprises ten β strands and twelve α helices arranged into two structural domains, the N-terminal open-face β-sandwich domain (N-lobe) and the C-terminal α/β-barrel domain (C-lobe) (Figure 1A). The secondary structure elements of the N-lobe consist of β1, β2, β3, β10, and α1–α5. The top layer of the sandwich is a four-stranded antiparallel β sheet consisting of β strands β1, β2, β3, and the end of the C-terminal β10 strand. Helices (α3–α5) form the second layer of the sandwich. The N-terminal domain is a triple-layered sandwich, as the N-terminal α4–α5 helices stacks on the top of helix α2. The N-terminal domain ends with the longest α helix, α5, which also marks the start of the α/β barrel. The C-terminal domain is an α/β barrel structure consisting of six β strands and seven α helices. *Ss*-QAPRTase forms a dimer via interaction between the N-lobe of one subunit and the C-lobe of the adjacent subunit (Figure 1B, C). The dimeric interface of *Ss*-QAPRTase buries approximately 3200 Å^2^ of the protein surface, which represents approximately 23.6% of the total accessible surface area of each subunit. The root mean square deviations (RMSDs) between corresponding C_α_ atoms of two subunits in the asymmetric unit is 0.44 Å. The active site residues are confined by the other dimer subunit and highly conserved in all QAPRTases (Figure S1). Dimerization is thought to be important in increasing substrate specificity and proper enzymatic function, as has been shown in all prior QAPRTase structures [7], [8], [15], [26], [28], [29].

**Figure 2:**
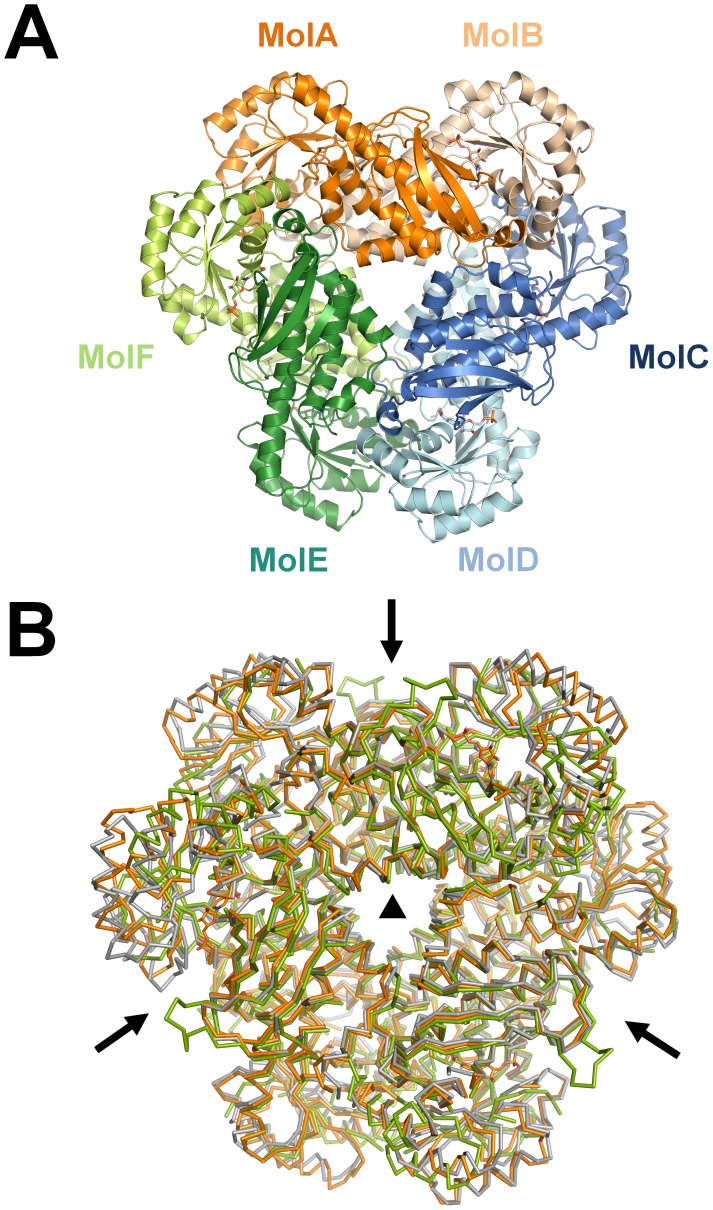
Hexamer organization of *Ss*-QAPRTase. (A) Hexamer structure. Dimer subunits were colored in orange, blue, and green. The lighter color indicates the other subunit of each dimer. (B) The superposition of hexameric QAPRTases from porcine (orange), human (gray, tartrate complex), and yeast (green, apo). The non-crystallographic two-fold axis of symmetry and crystallographic three-fold axis of symmetry are displayed as arrows and a triangle, respectively.

*Ss*-QAPRTase forms a hexamer organized as a trimer of dimers (Figure 2A). The three dimers of porcine QAPRTase form a hexamer with a triangular structure. The hexamer has approximate dimensions of 110×110×60 Å. The surface area of *Ss*-QAPRTase that is buried by the hexamer formation is approximately 2900 Å^2^ per dimer, which represents approximately 14% of the total surface area. Ionic and van der Waals interactions are the predominant contributors to the stabilization of the dimer and hexamer structure, respectively, rather than other non-covalent bonds. The *Ss*-QAPRTase structure has similar dimer-dimer interfaces and hexameric structure to those found eukaryotes, such as in the human [26] and yeast [28] enzymes; the RMSDs are 1.14 and 1.23 Å over 1006 and 904 aligned residues, respectively (Figure 2B, Table S2).

### NAMN Binding Site

**Figure 3:**
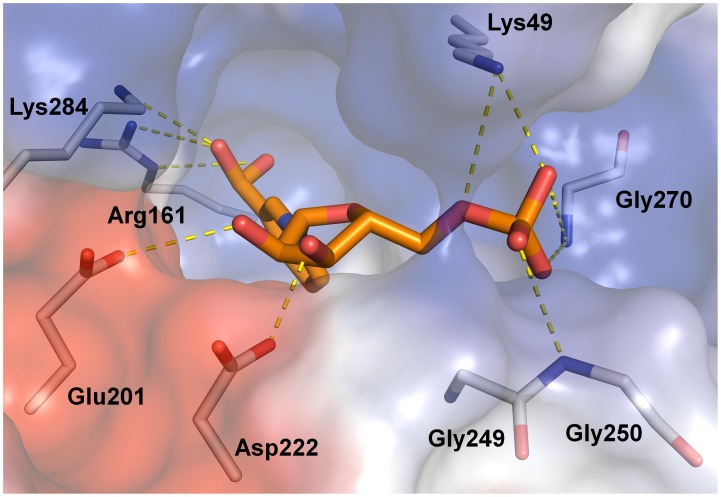
Surface representation of the Ss-QAPRTase active site (stereo view). The surface of the protein is colored based on the electrostatic potential. NAMN (orange) and residues in the active site are shown in sticks. The hydrogen bonds between a ligand and the active site residues are shown as dashed yellow lines.

In the *Ss*-QAPRTase–NAMN complex structure, the simulated annealing omit map for NAMN calculated with data extending to 2.1 Å showed clear electron density for the NAMN molecules bound to QAPRTase, with one molecule per QAPRTase subunit (Figure S4). The occupancy and RMSD of both NAMN molecules are 1.00 and 0.83 Å, respectively. The NAMN binding sites are located at the interfaces between the N-lobe of one subunit and the C-lobe of the other subunit in a dimer and are composed of residues from both subunits. The 3-carboxyl group of nicotinate moiety and the phosphate group occupy the basic pockets, whereas the hydroxyl groups of ribose ring make hydrogen bonds with the cavity consisting of Glu201 and Asp222 (Figure 3). The nicotinate ring of NAMN is located between the β4 and β5 strands, and the ribose phosphate groups extend across the barrel toward β strands β8 and β9 (Figure 1A). The ribose hydroxyl group oxygen atoms of NAMN are within hydrogen-bonding distance of Glu201 and Asp222. The phosphate group of NAMN makes hydrogen bonds with the main chain nitrogens of Gly249, Gly250, and Gly270 and the side chain nitrogens of Lys139, Asn223, and Gln274. In addition, Arg138, His160, Arg161, and Lys171 form a basic pocket and contribute to the hydrogen interaction with the 3-carboxyl group of the nicotinate moiety of NAMN.

**Figure 4:**
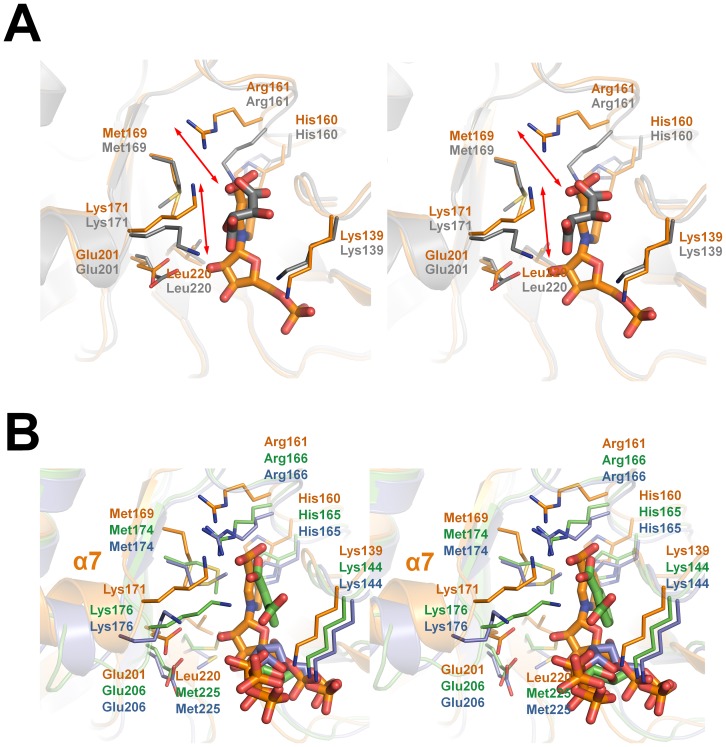
Structural comparison of *Ss*-QAPRTase–NAMN complex and eukaryotic enzymes. (A) Superposition of the *Ss*-QAPRTase–NAMN complex and the human enzyme in complex with tartrate (PDB ID: 2JBM), which mimics QUIN. The Porcine and human molecules are orange and gray, respectively. The movements of 3 Å are shown in two-sided red arrows. (B) Superposition of the NAMN binding sites of the porcine and yeast QAPRTases. The PRPP and PRPP/phthalate complexes of yeast QAPRTases (PDB ID: 3C2F and 3C2V) are colored in blue and green, respectively.

A structural comparison between the *Ss*-QAPRTase–NAMN complex and other eukaryotic enzymes is shown in Figure 4. Intriguingly, in the human QAPRTase–tartrate complex, Arg161, which has been reported to be a key residue for QUIN binding [26], moved approximately 3 Å away toward a tartrate molecule from its position in the *Ss*-QAPRTase–NAMN complex (Figure 4A). As the result, Arg161 in human enzyme binds not only to the carbonate moiety but also to the other side, mimicking a pyridine ring. Yeast QAPRTase-reactant complexes show a similar mode of ligand binding to *Ss*-QAPRTase, except that yeast QAPRTasein complex with phthalate and PRPP has a random coil rather than α7 (Figure 4B). In contrast, the complex structures of yeast QAPRTase with only one reactant (QUIN or PRPP) showed a conformation of α7 that is similar to the *Ss*-QAPRTase–NAMN complex. This suggests that the eukaryotic QAPRTase undergoes a rearrangement of the ionic interactions with the pyrophosphate moiety of PRPP and the carbonate of phthalate with the active site residues during the conversion from the reactant to the product. In addition, the ribose moieties of PRPP and/or phthalate in yeast QAPRTase complexes tilted approximately 120°, perhaps due to the conversion from the reactant to the product state (Figure 4B).

**Figure 5:**
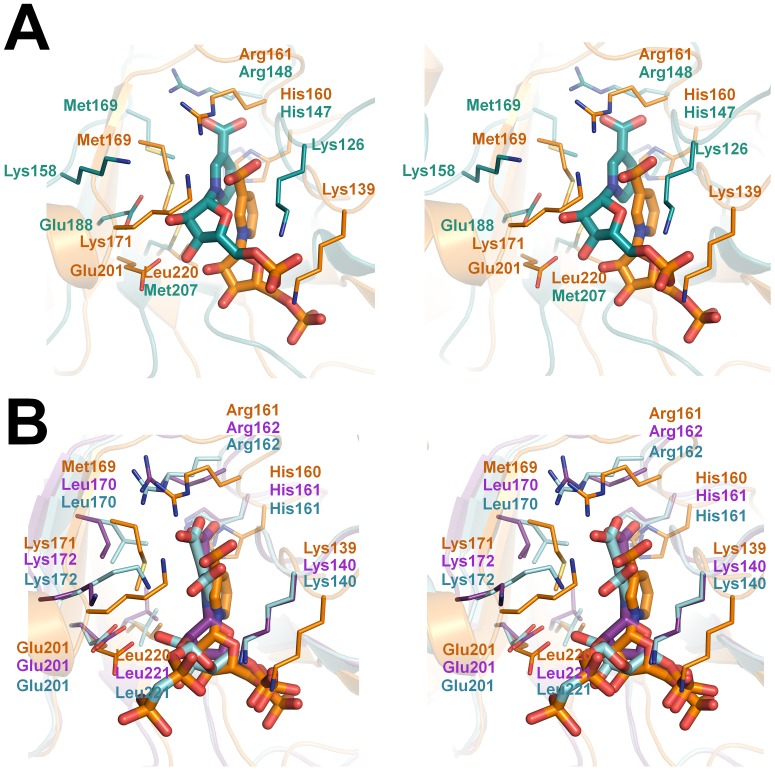
Comparing structures of *Ss*-QAPRTase with enzymes from pathogenic bacteria (stereo view). NAMN binding sites of porcine, *H. pylori* (A), and *M. tuberculosis* (B) QAPRTases are superposed. Porcine, *H. pylori* protein (PDB ID: 2B7Q), and *M. tuberculosis* enzymes in complex with NAMN (PDB ID: 1QPN) or phthalate/PRPP (PDB ID: 1QPR) are shown in orange, cyan, purple, and light blue, respectively.

In prokaryotes, the structures of QAPRTase in complex with NAMN have been determined from pathogenic bacteria, including *Helicobacter pylori* and *Mycobacterium tuberculosis*, which provide valuable comparisons with the *Ss*-QAPRTase–NAMN complex that may be useful in the development of new antibiotics (Figure 5). To accommodate the 3-carboxylate group of the nicotinate moiety of NAMN, *H*. *pylori* QAPRTase has 3 basic residues (Arg125, His 147, and Arg148) and *M. tuberculosis* QAPRTase has 4 residues (Arg136, Arg139, His161, and Arg162) in the deep active site pocket [8], [29]. Comparison of the NAMN and phthalate/PRPP complexes of *M. tuberculosis* QAPRTase shows that NAMNs in the both *M. tuberculosis* and *Ss* complexes occupy a different site from PRPP; NAMN in *M. tuberculosis* phthalate/PRPP complex is located inside the cavity instead of at the entrance (Figure 5B). The phosphate group of NAMN in *M. tuberculosis* structure is located in approximately the same position as that of PRPP [8]. Furthermore, the side chain of Lys171 in *Ss*-QAPRTase is located 3.5 Å closer than it is in the *M. tuberculosis* QAPRTase–NAMN complex, allowing it to make an ionic interaction with the hydroxyl group of nicotinate moiety of NAMN. Based on this structural difference, the generation of new antibiotic candidates with additional negative charges may be able to increase the selectivity for QAPRTases from pathogenic bacteria and avoid side effects by decreasing the affinity for the human enzyme. Introducing pyrophosphate moiety to the hydroxyl group of ribose ring in NAMN possibly makes additional ionic interaction with Lys172 in the *M. tuberculosis* QAPRTase instead of Lys171 in the human enzyme.

In summary, our crystal structure of porcine QAPRTase–NAMN complex is the first cocrystal structure of a mammalian QAPRTase with its reaction product. This structure may contribute to the rational design of selective inhibitors of high medical interest in a number of pathological conditions in humans.

## Supporting Information

Figure S1 **Gel filtration profile of the hexameric** ***Ss*** **-QAPRTases.** A superdex-200 16/60 column equilibrated in 20 mM HEPES–NaOH, pH 7.5, 100 mM KCl was used for gel filtration. Estimated molecular weights of the monomeric and hexameric QAPRTases are approximately 33 and 198 kDa, respectively. Fractions containing *Ss*-QAPRTases in gel filtration buffer showed molecular weight of 230 kDa.
(TIF)

Figure S2 **Multiple sequence alignment of the mammalian QAPRTases.** Total seven sequences were used aligned: Porcine-cDNA, porcine sequence from cDNA used in this study; Porcine-DB, porcine sequence derived from the raw DNA sequence in the database (NW_003534422.2); Human, human (NP_055113.2); Chimp, chimpanzee (JAA05453.1); Bovine, bovine (NP_001030523.1); Mouse, mouse (NP_598447.1); Bat, bat (ELK10952.1). Codes in parenthesis mean NCBI accession numbers. Region of the amino acid showing difference between Porcine-cDNA and Porcine-DB was highlighted in red box.
(TIF)

Figure S3 **Multiple sequence alignment of the QAPRTases used in structural comparison.** Mt and Hp indicate *Mycobacterium tuberculosis* and *Helicobacter pylori*, respectively. Highly conserved residues are shown in white characters with black background. Secondary structure elements are displayed above the sequences as red cylinders (α helices) and green arrows (β strands). Active site residues are highlighted by black circles (eukaryotes) and asterisks (prokaryotes). The N- and C-lobes are shaded in orange and blue, respectively.
(TIF)

Figure S4 **Electron density map of the NAMN.** The simulated annealing composite omit electron density map of the NAMN molecule in the *Ss*-QAPRTase–NAMN complex contoured at 1.0 σ.
(TIF)

Table S1 **Oligonucleotide primers used in this study.**
(DOC)

Table S2 **RMSDs (Å) of the hexameric structures of eukaryotic QAPRTases.**
(DOC)
