# Supplementary figures and images for: Crystal Structure of Sus scrofa Quinolinate Phosphoribosyltransferase in Complex with Nicotinate Mononucleotide
Source: PLoS One. 2013 Apr 23;8(4):e62027. doi: 10.1371/journal.pone.0062027 (PMC3633916; doi:10.1371/journal.pone.0062027)

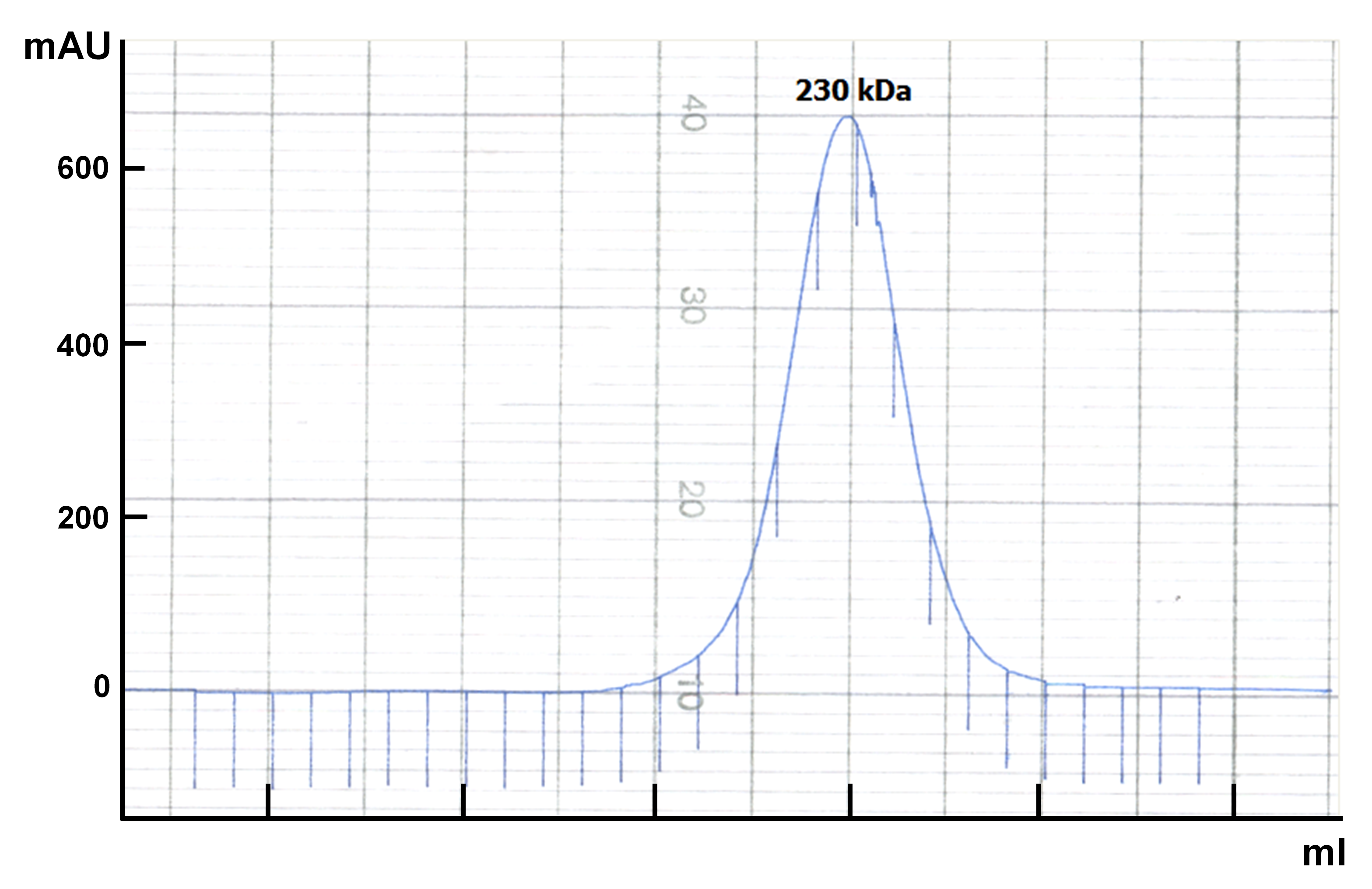

Supplement: Figure S1 — Gel filtration profile of the hexameric Ss -QAPRTases. A superdex-200 16/60 column equilibrated in 20 mM HEPES–NaOH, pH 7.5, 100 mM KCl was used for gel filtration. Estimated molecular weights of the monomeric and hexameric QAPRTases are approximately 33 and 198 kDa, respectively. Fractions containing Ss-QAPRTases in gel filtration buffer showed molecular weight of 230 kDa. (TIF) [file pone.0062027.s001.tif]

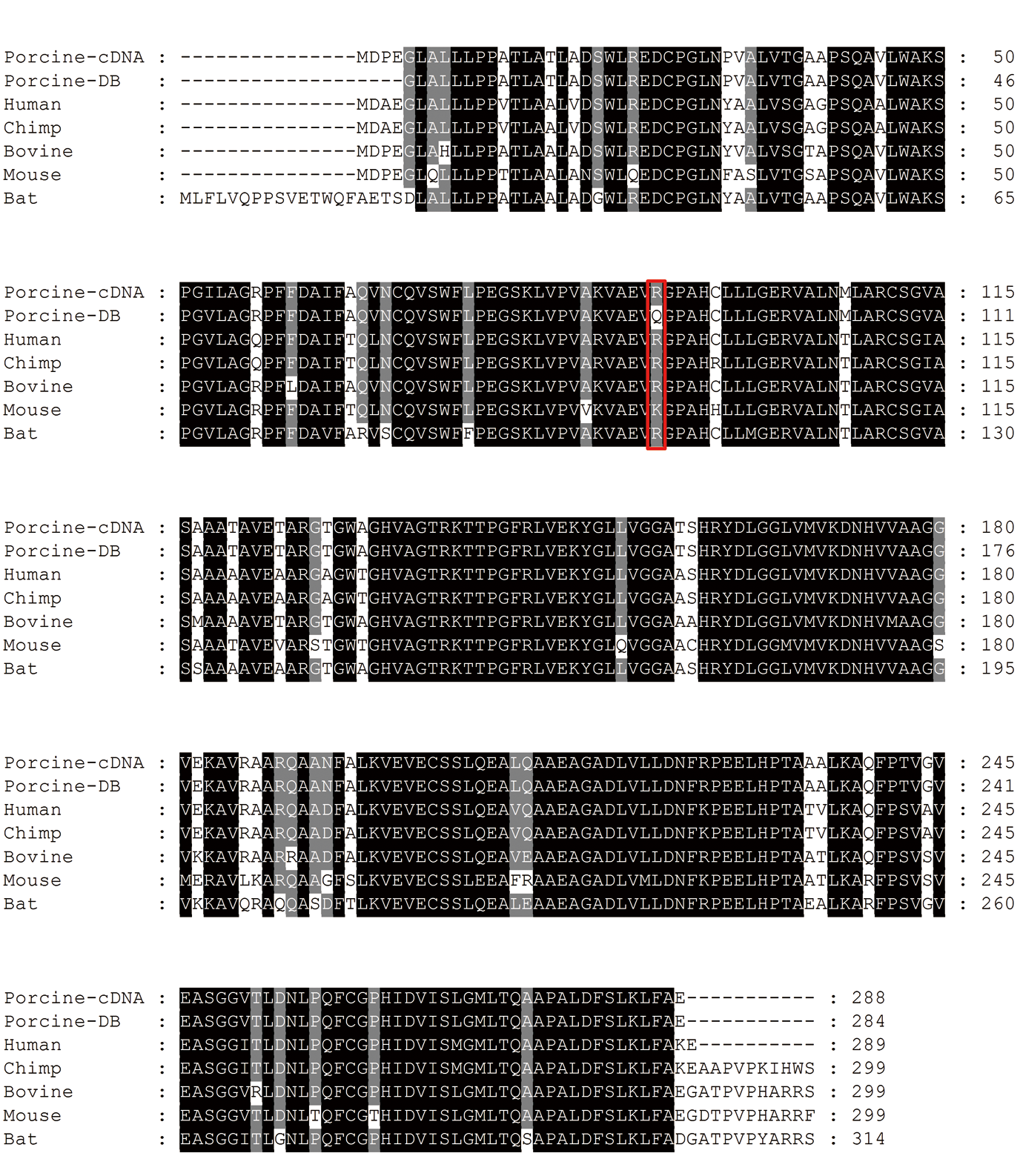

Supplement: Figure S2 — Multiple sequence alignment of the mammalian QAPRTases. Total seven sequences were used aligned: Porcine-cDNA, porcine sequence from cDNA used in this study; Porcine-DB, porcine sequence derived from the raw DNA sequence in the database (NW_003534422.2); Human, human (NP_055113.2); Chimp, chimpanzee (JAA05453.1); Bovine, bovine (NP_001030523.1); Mouse, mouse (NP_598447.1); Bat, bat (ELK10952.1). Codes in parenthesis mean NCBI accession numbers. Region of the amino acid showing difference between Porcine-cDNA and Porcine-DB was highlighted in red box. (TIF) [file pone.0062027.s002.tif]

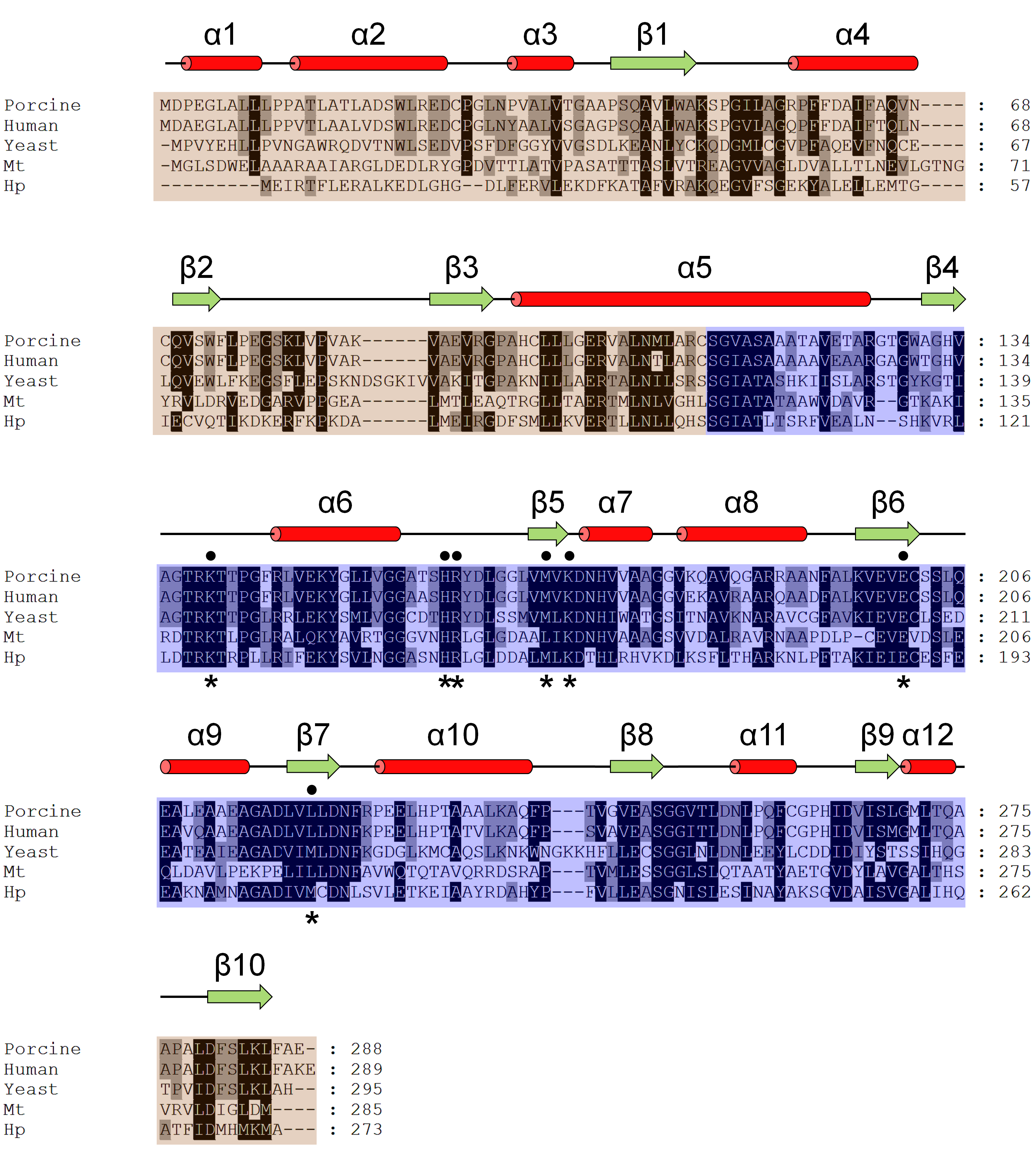

Supplement: Figure S3 — Multiple sequence alignment of the QAPRTases used in structural comparison. Mt and Hp indicate Mycobacterium tuberculosis and Helicobacter pylori, respectively. Highly conserved residues are shown in white characters with black background. Secondary structure elements are displayed above the sequences as red cylinders (α helices) and green arrows (β strands). Active site residues are highlighted by black circles (eukaryotes) and asterisks (prokaryotes). The N- and C-lobes are shaded in orange and blue, respectively. (TIF) [file pone.0062027.s003.tif]

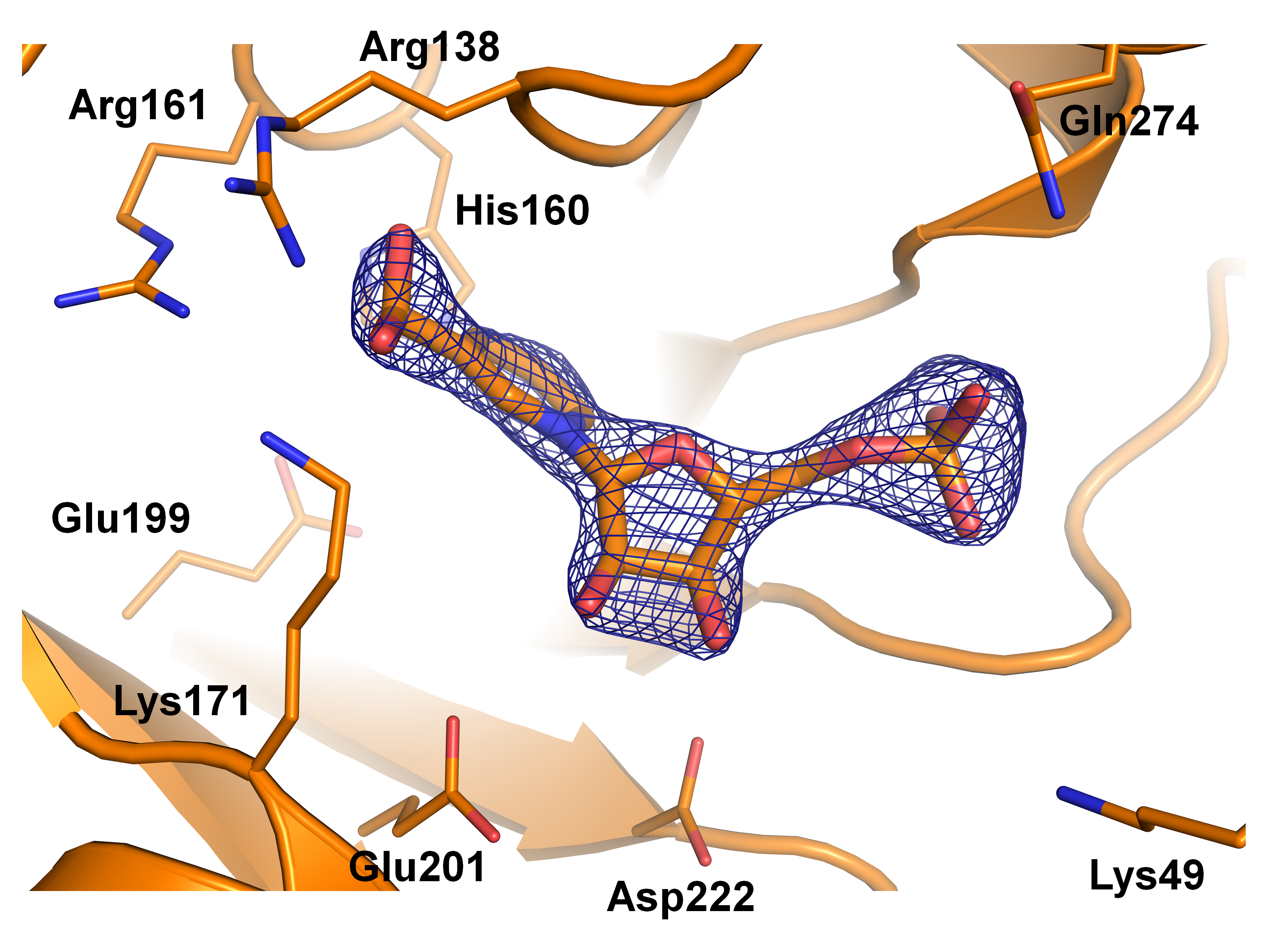

Supplement: Figure S4 — Electron density map of the NAMN. The simulated annealing composite omit electron density map of the NAMN molecule in the Ss-QAPRTase–NAMN complex contoured at 1.0 σ. (TIF) [file pone.0062027.s004.tif]
