# Supplementary material for: Crystal Structure of Sus scrofa Quinolinate Phosphoribosyltransferase in Complex with Nicotinate Mononucleotide
Source: PLoS One. 2013 Apr 23;8(4):e62027. doi: 10.1371/journal.pone.0062027 (PMC3633916; doi:10.1371/journal.pone.0062027)
Supplement: Table S1 — Oligonucleotide primers used in this study. (DOC) [file pone.0062027.s005.doc]

**Table S1. Oligonucleotide primers used in this study.**

| Primer name | Sequence^a^ |
| --- | --- |
|  |  |
| *Ss*-GAPDH-F^a^ | 5’-GATGACATCAAGAAGGTGGTG-3’ |
| *Ss* -GAPDH-R^a^ | 5’-CTCTTACTCCTTGGAGGCCATG-3’ |
| *Ss* -albumin-F^a^ | 5’-GCCCATATGAAGAGCATGTG-3’ |
| *Ss* -albumin-R^a^ | 5’-CCAACAGAGGCTTATCACAGC-3’ |
| *Ss*-QAPRTase-F^b^ | 5’-AG***GAATTC***CCATGGACCCTGAAGGCCTG-3’ |
| *Ss*-QAPRTase -R^c^ | 5’-CG***CTCGAG***TTATTCGGCAAACAGCTTGAGG-3’ |

^a^ GAPDH and albumin were used as positive controls

^b^ *Eco*RI site is indicate bold italics

^c^ *Xho*I site is indicate bold italics
