# Supplementary material for: Crystal Structure of Sus scrofa Quinolinate Phosphoribosyltransferase in Complex with Nicotinate Mononucleotide
Source: PLoS One. 2013 Apr 23;8(4):e62027. doi: 10.1371/journal.pone.0062027 (PMC3633916; doi:10.1371/journal.pone.0062027)
Supplement: Table S2 — RMSDs (Å) of the hexameric structures of eukaryotic QAPRTases. (DOC) [file pone.0062027.s006.doc]

**Table S2. RMSDs (Å) of the hexameric structures of eukaryotic QAPRTases.**

Porcine (obtained structure), human (apo, PDB ID 3LAR; tartrate complex, PDB ID 2JBM), yeast (apo, PDB ID 3C2E; PRPP complex, PDB ID 3C2F; phthalate/PRPP complex, PDB ID 3C2V) QAPRTases were used for calculating RMSDs.

|  | **Porcine**  **NAMN** | **Human**  **Apo** | **Human**  **Tartrate** | **Yeast**  **Apo** | **Yeast**  **PRPP** | **Yeast**  **Phthalate**  **PRPP** |
| --- | --- | --- | --- | --- | --- | --- |
| **Porcine**  **NAMN** |  | 1.14 | 0.57 | 1.23 | 1.28 | 1.25 |
| **Human**  **Apo** |  |  | 1.16 | 1.22 | 1.22 | 1.26 |
| **Human**  **Tartrate** |  |  |  | 1.16 | 1.22 | 1.18 |
| **Yeast**  **Apo** |  |  |  |  | 0.42 | 0.46 |
| **Yeast**  **PRPP** |  |  |  |  |  | 0.61 |
| **Yeast**  **Phthalate**  **PRPP** |  |  |  |  |  |  |
